# Autophagy and bacterial clearance: a not so clear picture

**DOI:** 10.1111/cmi.12063

**Published:** 2012-12-02

**Authors:** Serge Mostowy

**Affiliations:** Section of Microbiology, MRC Centre for Molecular Bacteriology and Infection, Imperial College LondonArmstrong Road, London, SW7 2AZ, UK

## Abstract

Autophagy, an intracellular degradation process highly conserved from yeast to humans, is viewed as an important defence mechanism to clear intracellular bacteria. However, recent work has shown that autophagy may have different roles during different bacterial infections that restrict bacterial replication (antibacterial autophagy), act in cell autonomous signalling (non-bacterial autophagy) or support bacterial replication (pro-bacterial autophagy). This review will focus on newfound interactions of autophagy and pathogenic bacteria, highlighting that, in addition to delivering bacteria to the lysosome, autophagy responding to bacterial invasion may have a much broader role in mediating disease outcome.

## Introduction

Autophagy is an intracellular process delivering cytoplasmic material to the lysosome for degradation. The cellular events of this ancient and highly conserved process have been well characterized: cytoplasmic material is enclosed by an isolation membrane, called a phagophore, which elongates to form a double-membraned vacuole, called an autophagosome; the autophagosome fuses with the lysosome to form an autolysosome and degrade the enclosed material. In this way, autophagy acts as a cytoplasmic quality control mechanism, eliminating protein aggregates, damaged organelles and intracellular microbes to maintain cellular homeostasis (Levine *et al*., 2011; Mizushima and Komatsu, 2011). Autophagy involves the assembly of 36 autophagy-related (ATG) proteins into complexes that are essential for different steps of autophagosome formation: the ATG1-UNC-51-like kinase (ULK) complex triggers autophagy, the class III phosphatidylinositol 3 (PI3) kinase complex generates PI3P (an essential lipid component of autophagosomes), the ATG12–ATG5–ATG16L1 ubiquitin-like conjugation system mediates formation and elongation of the autophagosome and the ATG8 ubiquitin-like conjugation system mediates closure of the phagophore (Mizushima *et al*., 2011). Despite identification of the ATG proteins and complexes, the molecular mechanisms and signalling networks controlling autophagosome formation have not yet been fully defined (Mizushima *et al*., 2011; Hamai and Codogno, 2012). Of particular interest is the source of membrane for autophagosome biogenesis, which enables a remarkable plasticity in determining the location and size of autophagosome formation (Tooze and Yoshimori, 2010).

When autophagy was discovered over 50 years ago it was considered a general, non-selective degradative pathway activated by nutrient limitation. However, it has been increasingly recognized that autophagosomes may also degrade cytosolic material, such as intracellular bacteria, in a selective manner. While the exact mechanism of bacterial recognition by autophagy remains unknown, the best-characterized process involves ubiquitination (Shaid *et al*., 2012). Autophagy receptors, such as p62 (sequestosome 1 or SQSTM1), NBR1 (neighbour of BRCA1 gene 1), NDP52 (nuclear dot protein, 52 kDa) and OPTN (optineurin), are pattern recognition receptors, called sequestosome 1/p62-like receptors (SLRs), that recognize ubiquitinated substrates and recruit membranes for autophagosome formation through their interaction with ATG8 family proteins (Deretic, 2012). Over the past 10 years, autophagy has been viewed as a crucial host cell response to bacterial invasion by delivering intracellular pathogens to the lysosome. However, newfound interactions of autophagy and pathogenic bacteria has revealed that autophagy may have different roles during different bacterial infections that, in addition to bacterial clearance, co-ordinate cell autonomous signalling and in some cases promote bacterial replication. As a result, autophagy can no longer be viewed as strictly antibacterial, and the therapeutic potential of autophagy to resolve bacterial infection remains to be fully defined.

## Interactions of autophagy and pathogenic bacteria

**Fig. 1:**
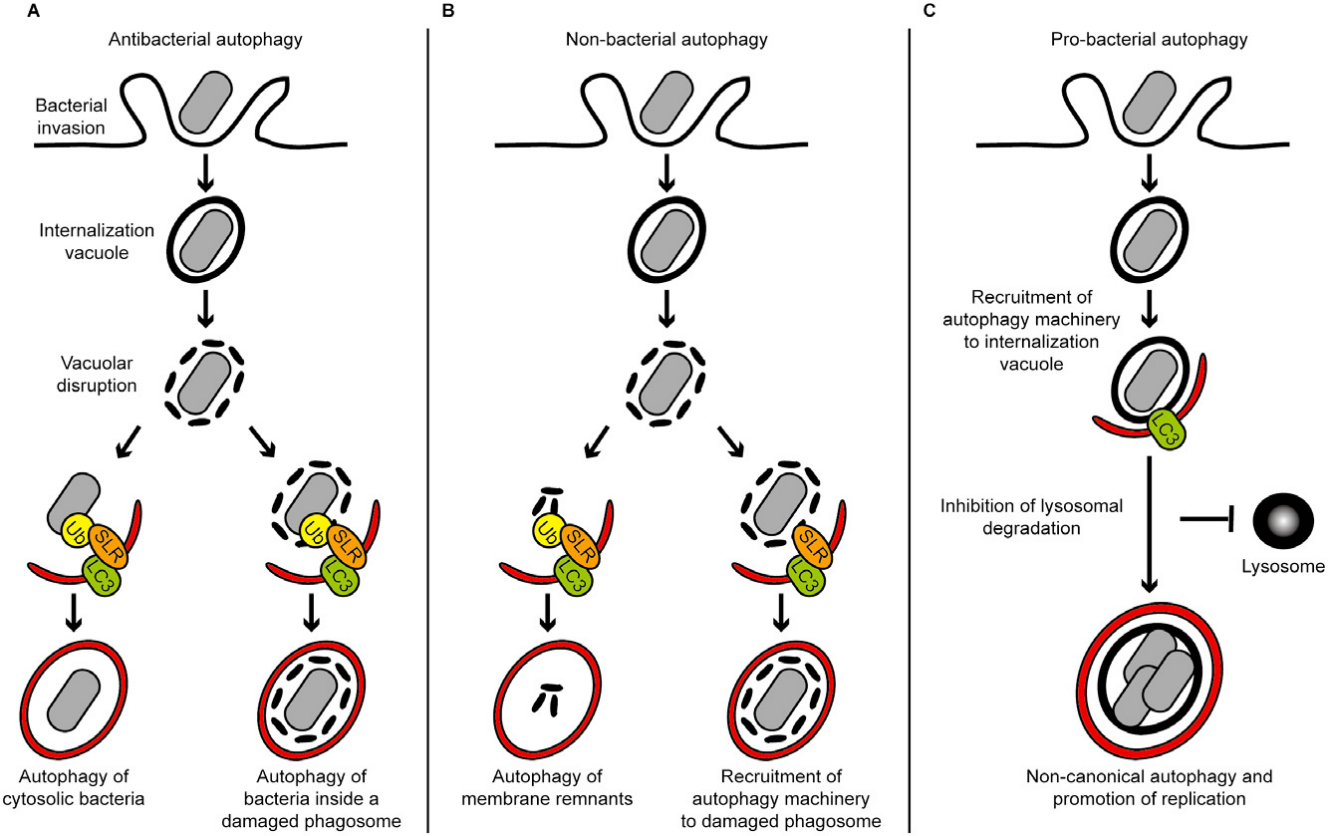
Different autophagy pathways triggered by bacterial invasion. A. Antibacterial autophagy. After entry into host cells, bacteria are localized inside an internalization vacuole. Upon vacuolar disruption, autophagy may recognize ubiquitination signals and intracellular pathogens located (left) in the cytosol (e.g. *L. monocytogenes*, *S. flexneri*, *S.* Typhimurium) and (right) inside a damaged internalization vacuole (e.g. *M. tuberculosis*). In both cases, the enclosed bacterium is delivered to the lysosome for degradation. B. Non-bacterial autophagy. Autophagy may be targeted against cellular disturbances arising from the bacterial invasion process, such as membrane damaged from bacterial entry or vacuolar disruption. (Left) Damaged membrane, and inflammasome components localized to damaged membrane, may be ubiquitinated and targeted to autophagy. (Right) Damaged membrane can also be recognized for autophagy by non-ubiquitin signals (e.g. NDP52–galectin 8). In both cases, non-bacterial autophagy may trigger cell autonomous signalling and influence bacterial replication. C. Pro-bacterial autophagy. Some internalized bacteria (e.g. *S. aureus*, *B. abortus*) may recruit a subset of the autophagy machinery and create a replicative niche inside an autophagosome-like vacuole. These bacteria subvert the autophagy machinery to avoid degradation in a lysosomal compartment and support bacterial replication. Ub, ubiquitin; SLR, autophagy receptor (e.g. p62, NDP52); LC3, ATG8 family proteins.

Intracellular pathogens can be uptaken passively by macrophages (e.g. mycobacteria) or can actively invade epithelial cells (e.g. *Listeria*, *Shigella* or *Salmonella*). After internalization, bacteria are either transiently or definitively localized within an internalization vacuole, called a phagosome. Some pathogens escape from the phagosome to the cytosol and avoid destruction in phagolysosomes (e.g. *Listeria* or *Shigella*), whereas other pathogens interfere with phagolysosome biogenesis and form replicative vacuoles (e.g. *Salmonella* or mycobacteria). Pioneering studies have shown that autophagy can degrade intracellular pathogens located both in the cytosol (Ogawa *et al*., 2005; Yoshikawa *et al*., 2009) and inside the phagosome (Gutierrez *et al*., 2004; Birmingham *et al*., 2006). However, it now appears that autophagy has different roles during different bacterial infections that may restrict bacterial replication (antibacterial autophagy), act in cell autonomous signalling (non-bacterial autophagy) or support bacterial replication (pro-bacterial autophagy) (Fig. 1). Here I focus on novel autophagy–bacteria interactions to illustrate these alternative outcomes, suggesting that autophagy should be viewed as having a much broader role in the host response to infection than only delivering bacteria to the lysosome.

### Antibacterial autophagy and pathogen clearance

Studies using *Listeria monocytogenes*, *Shigella flexneri*, *Salmonella* Typhimurium and *Mycobacterium tuberculosis* have independently highlighted autophagy in the restriction of bacterial replication. A theme emerging from these studies is that bacteria inadvertently exposed to the host cytosol are cleared by autophagy, whereas bacteria intentionally accessing the host cytosol for replication have evolved mechanisms to avoid recognition by autophagy (Randow and Münz, 2012).

*Listeria monocytogenes*, a Gram-positive bacterium, survives intracellularly by escaping from phagosomes using LLO, a pore-forming cytotoxin (Cossart, 2011). In the cytosol, *Listeria* uses its surface-expressed ActA protein to directly recruit the Arp2/3 complex and form actin tails for motility (Haglund and Welch, 2011). At the same time, ActA prevents ubiquitination and the recruitment of autophagy receptors (p62 and NDP52) to *Listeria* (Yoshikawa *et al*., 2009; Mostowy *et al*., 2011). In the absence of ActA, InlK is a *Listeria* surface protein (which is only expressed *in vivo*) that can recruit the major vault protein (MVP) and also prevent autophagic recognition of bacteria (Dortet *et al*., 2011). These observations suggest that bacterial surface proteins inhibit recruitment of the autophagy machinery, or that host proteins recruited by *Listeria* disguise bacteria from autophagic recognition. In either case, ubiquitination and autophagic clearance of *Listeria* requires the absence of ActA and InlK, and benefits from multiple autophagy receptors. Strikingly, *Listeria* is recognized by autophagy in the absence of the actin or septin cytoskeleton (Mostowy and Cossart, 2012a), suggesting that autophagic degradation of *Listeria* and *Shigella* does not strictly require the same molecular machinery (Mostowy *et al*., 2010; 2011).

**Fig. 2:**
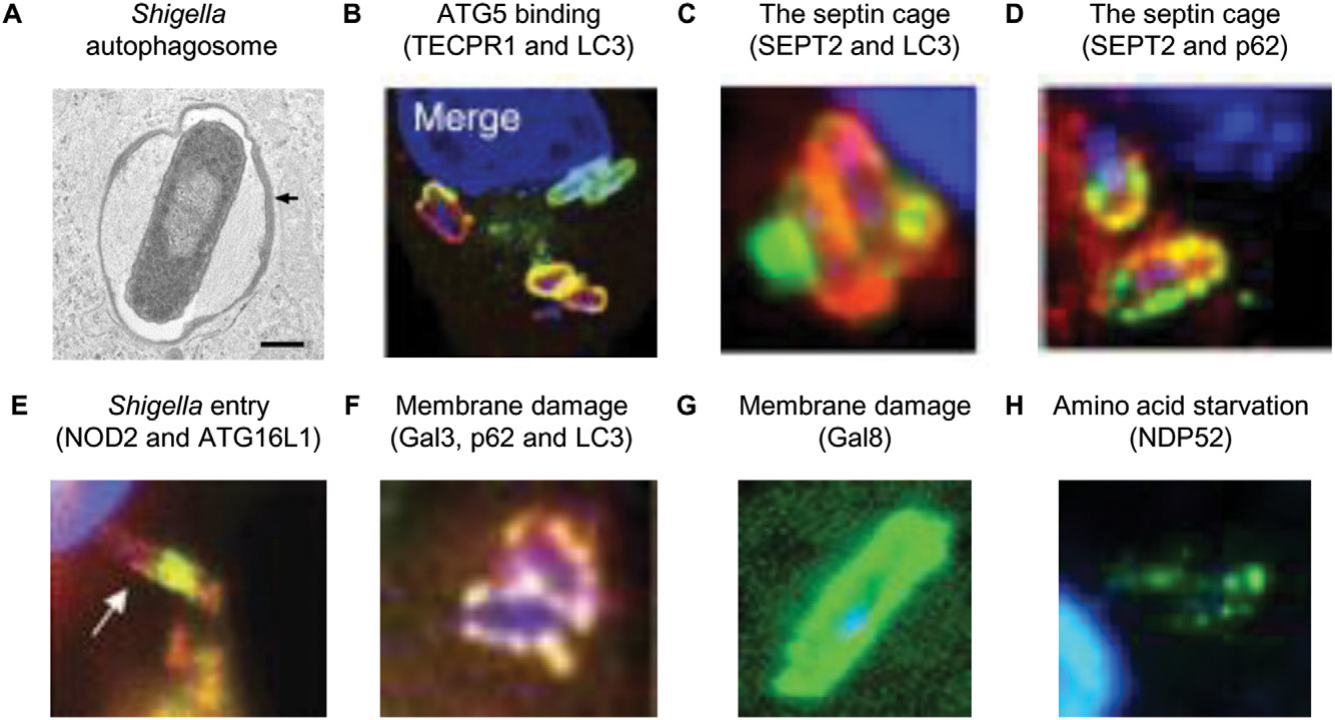
The *Shigella* paradigm. Several autophagy pathways are recruited to *S. flexneri*, and autophagy (using non-mutually exclusive and parallel recognition events) may have different roles during *Shigella* infection. It will be important to identify unique markers for the different autophagy pathways triggered by *Shigella*, and to define their specific roles in pathogen clearance. A. EM image showing LC3-positive, double membrane surrounding cytosolic *Shigella* (Ogawa *et al*., 2005). B. TECPR1 (red) binds to ATG5 and localizes with LC3 (green) around cytosolic *Shigella* in the absence of ubiquitin (Ogawa *et al*., 2011). C. The septin cage (SEPT2, red) entraps cytosolic *Shigella* and targets bacteria to autophagy (LC3, green) (Mostowy *et al*., 2010). D. Recruitment of the septin cage (SEPT2, red) to cytosolic *Shigella* is interdependent with recruitment of ubiquitin and autophagy receptors (p62, green) (Mostowy *et al*., 2010). E. NOD proteins (NOD2, green) and ATG16L1 (red) are recruited to the *Shigella* entry site and promote autophagy (Travassos *et al*., 2010). F. Galectin 3 (red), a marker for damaged membrane, localizes with autophagy receptors [p62 (here in cyan), NBR1 and NDP52] and LC3 (green) around *Shigella* (Dupont *et al*., 2009; Ligeon *et al*., 2011). G. Galectin 8 (green), a marker for damaged membrane, surrounding *Shigella* may recruit NDP52 in the absence of ubiquitin (Thurston *et al*., 2012). H. Membrane damage, labelled by NDP52 (green), around cytosolic *Shigella* causes intracellular amino acid starvation (Tattoli *et al*., 2012).

*Shigella flexneri* is a Gram-negative pathogen that escapes from its internalization vacuole. Once in the cytosol, *Shigella* uses its surface-expressed IcsA protein to recruit N-WASP and the Arp2/3 complex to form actin tails for motility (Haglund and Welch, 2011). Autophagy of *Shigella* is triggered by ATG5 recognition of IcsA (Ogawa *et al*., 2005), and is mediated by TECPR1, a Tectonin domain-containing protein, which binds to ATG5 and promotes autophagosome–lysosome fusion (Ogawa *et al*., 2011; Chen *et al*., 2012). To restrict bacterial motility and autophagy escape, septins are guanosine triphosphate (GTP) binding proteins recruited to sites of IcsA-induced actin polymerization, and form cage-like structures with ubiquitinated proteins and autophagy receptors (p62, NBR1 and NDP52) around actin-polymerizing bacterium (Mostowy *et al*., 2010; 2011). *Shigella* thus provides an example of a bacteria targeted to autophagosomes via a combination of ubiquitin-independent (i.e. recognized by ATG5–TECPR1) and ubiquitinated (i.e. recognized by autophagy receptors) signals (Fig. 2). As a countermeasure to avoid autophagy, *Shigella* may express IcsB, a type III secretion system (T3SS) effector, which competitively binds IcsA to inhibit ATG5 binding, TECPR1 recruitment and septin cage formation (Ogawa *et al*., 2005; 2011; Mostowy *et al*., 2010; 2011). *Shigella* may also express another T3SS effector, VirA, to counteract antibacterial autophagy. VirA exhibits GTPase-activating protein (GAP) activity, and manipulation of Rab1 GTPase function by VirA mediates suppression of autophagy, contributing to *Shigella* intracellular survival (Dong *et al*., 2012). Taken together, *Shigella* clearance by autophagy may benefit from the absence of IcsB and VirA, and requires ATG5–TECPR1 binding, multiple autophagy receptors, actin polymerization and septin assembly.

After internalization into host cells, *S.* Typhimurium, a Gram-negative pathogen, mostly resides and replicates within a modified phagosomal compartment called the *Salmonella*-containing vacuole (SCV). However, a fraction of *Salmonella* can become cytosolic and surrounded by ubiquitin (Perrin *et al*., 2004). Autophagy receptors discovered to target ubiquitinated *Salmonella* to autophagic degradation include p62 (Zheng *et al*., 2009), NDP52 (Thurston *et al*., 2009) and OPTN (Wild *et al*., 2011). To efficiently clear *Salmonella*, OPTN is phosphorylated by TANK binding kinase 1 (TBK1), an IKK-related kinase responsible for autophagosome maturation (Wild *et al*., 2011). Why multiple autophagy receptors are important for bacterial autophagy remains unknown. Recent work has shown that NDP52, unlike p62, directly interacts with the rarely investigated LC3C (autophagy studies commonly use LC3B to represent ATG8 family proteins), and the selectivity of NDP52 for LC3C is critical for anti-*Salmonella* autophagy (von Muhlinen *et al*., 2012). The interaction between autophagy receptors and ATG8 family proteins reveals an unexplored specificity underlying selective autophagy, and also suggests a hierarchical recruitment of different ATG8 family proteins (i.e. LC3C is recruited first by NDP52, followed by the recruitment of other ATG8 family proteins including LC3B) that may explain the distinct membrane domains recruited to the *Salmonella* autophagosome (Cemma *et al*., 2011; Wild *et al*., 2011; Thurston *et al*., 2012). In sum, autophagic restriction of cytosolic, ubiquitinated *Salmonella* requires multiple autophagy receptors and the direct interaction of NDP52 with LC3C. *Salmonella* may also be targeted to autophagosomes via ubiquitin-independent signals. Network analysis has identified TOCA-1 (formin binding protein 1-like or FNBP1L), a transducer of Cdc42-dependent actin assembly, as an ATG3-interacting partner in *Salmonella*-infected cells (Huett *et al*., 2009). Via recruitment of ATG3, and not ubiquitination signals, TOCA-1 activity promotes autophagosome biogenesis and mediates anti-*Salmonella* autophagy. Interestingly, in the case of *Shigella*, TOCA-1 is required for efficient N-WASP-mediated actin tail polymerization (Leung *et al*., 2008). Whether or not TOCA-1 is required for *Shigella*–septin cage formation and autophagy, also dependent on N-WASP activity (Mostowy *et al*., 2010), has yet to be tested.

*Mycobacterium tuberculosis*, the causative agent of human tuberculosis, is a vacuolar pathogen that survives within macrophages by arresting phagosomal maturation. A variety of studies have shown that the induction of autophagy by starvation, inhibition of mTOR (mammalian target of rapamycin, a suppressor of autophagy), vitamin D and interferon-gamma (IFNγ) may help restrict mycobacterial replication (Deretic *et al*., 2009; Fabri *et al*., 2011). p62 appears to be crucial for this process, and provides mycobacterial autophagolysosomes (phagosomes surrounded by an LC3-positive double membrane) with enhanced antimicrobial capacities relative to conventional phagolysosomes (Ponpuak *et al*., 2010). To efficiently detect and eliminate mycobacteria, p62 is phosphorylated by TBK1 (Pilli *et al*., 2012), yet how *M. tuberculosis* triggers autophagy from within the phagosome has been a puzzling issue. Recent work has shown that membrane permeabilization by the mycobacterial ESX-1 secretion system enables ubiquitin-mediated autophagy to recognize phagosomal *M. tuberculosis* (Watson *et al*., 2012). Recognition of bacterial DNA by the adaptor STING (stimulator of interferon genes) is required for ubiquitination of bacteria, and delivery of ubiquitinated *M. tuberculosis* to autophagolysosomes requires p62 and NDP52. Strikingly, ATG5-deficient mice are highly susceptible to *M. tuberculosis* infection, highlighting autophagy as a major determinant of host resistance to *M. tuberculosis* infection *in vivo* (Castillo *et al*., 2012; Watson *et al*., 2012). These studies have shown that while delivery of *M. tuberculosis* to the lysosome may have a direct role in acute bacterial restriction, autophagy may have additional roles in overall control by suppressing bacterial growth and by preventing excessive inflammation.

### Non-bacterial autophagy and cell autonomous responses to bacterial invasion

Whereas autophagic delivery of bacteria to the lysosome can have a direct role in bacterial restriction, autophagy may also control infection via regulation of cell autonomous immune responses. In this case, work has shown that autophagic recognition of bacterial invasion is not targeted against bacteria *per se*, and is targeted against cellular disturbances arising from the invasion process, such as membrane damage. From the examples of *Shigella* and *Salmonella*, recognition of bacterial invasion by non-bacterial autophagy may act in immune signalling and influence bacterial replication.

Several studies have highlighted a central role for autophagy in regulating the response to membrane damaged by invasive bacteria (Fig. 2). Studies using *Shigella* have shown that NOD proteins (pattern recognition receptors distinct from SLRs) are recruited with ATG16L1 to the plasma membrane at the site of bacterial entry and trigger autophagy (Travassos *et al*., 2010). In the cytosol of infected cells, membrane remnants induced by invading *Shigella* are ubiquitinated and recognized by p62, NBR1 and NDP52 for delivery to autophagosomes (Dupont *et al*., 2009; Ligeon *et al*., 2011). Inflammasome components, localized to damaged membranes, are also ubiquitinated and recognized by p62 for autophagy (Shi *et al*., 2012). Thus, autophagy accompanies membrane damage and inflammasome activation to control the immune response by eliminating membrane and active inflammasomes. Recent work using *Salmonella*, and corroborated using *Shigella* and *Listeria*, has revealed that NDP52 can also be recruited to damaged vacuoles marked by galectin 8, a cytosolic β-galactoside binding lectin, independently of ubiquitin (Thurston *et al*., 2012). Although NDP52–galectin 8 interactions presumably serve to restrict bacterial replication, they may also help to recruit LC3-positive membrane and repair damaged vacuoles. In agreement with autophagy playing a role in membrane repair, membrane fusion has been shown to act as a danger signal that, when recognized by STING, also triggers cell autonomous immune responses and cell survival (Holm *et al*., 2012).

The panoply of events that follow membrane damage and trigger non-bacterial autophagy is starting to emerge. The recognition of membrane damage may proceed with the recruitment of diacylglycerol (DAG), and work using *Salmonella* has shown that DAG-dependent signalling triggers autophagy and contributes to pathogen clearance (Shahnazari *et al*., 2010; Cemma and Brumell, 2012). Host membrane damage by *Salmonella* and *Shigella* also triggers intracellular amino acid starvation, itself a potent stimulus of autophagy (Tattoli *et al*., 2012). Indeed, pathogen-induced amino acid starvation dampens the activity of mTOR, a serine/threonine protein kinase that regulates a wide range of cellular responses including autophagy (Laplante and Sabatini, 2012). Thus, amino acid starvation is a newfound element of the immune response to intracellular bacteria.

The precise role of non-bacterial autophagy in the control of bacterial replication is not fully defined. At least in the case of *Salmonella*, non-bacterial autophagy triggered by the p62-mediated recognition of cytosolic, ubiquitinated structures which accompany infection has been shown to restrict bacterial replication (Mesquita *et al*., 2012). However, *Salmonella* can inhibit the selective autophagy of ubiquitinated structures by expressing SseL, a T3SS effector which acts as a deubiquitinase, and SseL activity lowers autophagic flux and promotes bacterial replication. This infection scenario is similar to autophagy triggered by *Mycobacterium marinum*, an intracellular pathogen that causes tuberculosis-like disease in ectotherms. In cells infected with *M. marinum*, cytosolic aggregates comprised of host and bacterial membrane remnants are ubiquitinated and targeted to autophagy (Collins *et al*., 2009). However, the role of non-bacterial autophagy in response to these ubiquitinated structures in *M. marinum* replication is not yet known.

### Pro-bacterial autophagy and the support of bacterial replication

Canonical autophagy is dependent on the hierarchical and co-ordinated recruitment of ATG proteins to the phagophore, to form and elongate an autophagosome that will fuse with the lysosome. By contrast, non-canonical autophagy may not require all of the autophagy machinery to form autophagosome-like vacuoles, and can be recognized when a subset of ATG proteins are recruited to an already-existing membrane (Mostowy and Cossart, 2012b). A well-characterized example of non-canonical autophagy is LC3-associated phagocytosis (LAP), a process in which phagosomes containing bacteria can recruit LC3 to promote phagosome maturation and degradation of cargo (Sanjuan *et al*., 2007). However, new evidence suggests that non-canonical autophagy can benefit the infection of some pathogens, including *L. monocytogenes*, *Staphylococcus aureus*, *Brucella abortus* and uropathogenic *Escherichia coli*.

While intracellular *L. monocytogenes* can evade autophagy in the cytosol via expression of ActA or InlK, a subpopulation may co-opt autophagy machinery and slowly replicate inside vacuoles called SLAPs (spacious *Listeria*-containing phagosomes) (Birmingham *et al*., 2008). SLAP formation occurs via the LAP pathway and requires dampened activity of LLO to damage membrane and inhibit fusion with the lysosome (Cemma and Brumell, 2012). In this way, SLAPs may enable chronic bacterial infection.

*S. aureus* is a Gram-positive bacterium that can invade cells and replicate in autophagosome-like vacuoles that colocalize with LC3 (Schnaith *et al*., 2007). Hla (α-haemolysin), a pore-forming toxin secreted by *S. aureus*, is required for the recruitment of LC3, suggesting the recruitment of autophagy components to membrane damage mediated by Hla (Mestre *et al*., 2010). Hla-induced autophagy requires ATG5, but does not require Beclin1 (ATG6) nor PI3 kinase activity, highlighting the benefit of a non-canonical autophagy pathway for *S. aureus* replication.

After entry into host cells, *B. abortus*, a Gram-negative intracellular pathogen, establishes a replicative compartment called *Brucella*-containing vacuoles (BCVs). To promote infection, *Brucella* may co-opt autophagosome initiation factors ATG1 (ULK1), Beclin1 and ATG14 to convert BCVs into autophagosome-like compartments called autophagic BCVs (aBCVs) (Starr *et al*., 2012). A non-canonical autophagy pathway here promotes bacterial replication and survival since autophagosome elongation factors ATG4B, ATG5, ATG7, LC3B and ATG16L1 are not required for biogenesis of aBCVs. Considering recent evidence showing that *Legionella pneumophila*, a Gram-negative intracellular pathogen that may exploit similar components of the autophagy machinery as *Brucella* (Mostowy and Cossart, 2012b), has evolved a mechanism to inhibit autophagy using the type IV secretion system (T4SS) effector RavZ to irreversibly inactivate ATG8 (Choy *et al*., 2012), it is tempting to speculate that the *Brucella* T4SS may also co-ordinate aBCV formation.

Whereas evidence that *Shigella* or *Salmonella* can exploit non-canonical autophagy for replication has not been obtained, recent work has shown that ATG16L1 deficiency confers host protection *in vivo* against infection from another Gram-negative pathogen, uropathogenic *Escherichia coli* (UPEC) (C. Wang *et al*., 2012). How UPEC may co-opt ATG16L and avoid autophagic degradation remains to be fully determined.

## Perspectives

There has been much recent progress in understanding autophagy and how it controls the fate of intracellular bacteria, highlighting different roles for autophagy during different bacterial infections. From the examples of *Listeria*, *Shigella*, *Salmonella* and mycobacteria, antibacterial autophagy may restrict bacterial replication, and non-bacterial autophagy triggered by membrane damage may have a critical role in cell autonomous immune responses. By contrast, from the examples of *Staphyloccus*, *Brucella* and UPEC, some bacterial pathogens may benefit from pro-bacterial, non-canonical autophagy pathways that support bacterial replication. These alternative autophagy–bacteria interactions strongly suggest that autophagy does more than deliver bacteria to the lysosome, and should be recognized for a much broader role in the response to infection. As a result, more research is required to clarify the therapeutic potential of autophagy (Box 1). Understanding these issues may suggest the development of new strategies aimed at bacterial infection, and possibly other infectious, autoimmune and inflammatory disease states that also implicate autophagy (Levine *et al*., 2011; Mizushima and Komatsu, 2011; Rubinsztein *et al*., 2012).

**Box 1. Critical issues in autophagy–bacteria interactions**

- What is the relative importance of ubiquitin and non-ubiquitin signals in pathogen clearance? Can the recognition of bacteria by autophagy be increased/altered to favour bacterial degradation?
- Components of the cytoskeleton/membrane interface [e.g. septins, TOCA-1 and vimentin (R.C. Wang *et al*., 2012)] may be key mediators of autophagosome formation. Can these components be used to enhance recruitment of the autophagy machinery to bacteria?
- SLRs have been specifically implicated in innate immunity, but may have a more general role in selective autophagy (Gibbings *et al*., 2012; Tumbarello *et al*., 2012). Are there SLRs exclusively dedicated to pathogen clearance?
- *Saccharomyces cerevisiae* encodes only a single ATG8 gene, yet humans and other animals encode multiple ATG8 genes belonging to different subfamilies, i.e. LC3s or GABARAPs. Does the expansion of ATG8 genes reflect another layer of specificity underlying bacterial autophagy?
- What is the source of membrane for antibacterial autophagy? Can the initial sequestering membrane be used to regulate autophagic activity (e.g. control autophagosome location and size)?
- Under what circumstance can cytokines (e.g. TNFα, IFNγ, IL-1β) and other physiological stimuli (e.g. rapamycin, amino acid starvation) be applied to induce autophagic degradation of bacteria?
- Can non-canonical autophagy pathways be manipulated to favour bacterial degradation? Forcing aspects of the ATG-dependent machineries to overcome autophagy blockage (e.g. targeted delivery of ATG8 family proteins) may transform non-canonical autophagy into canonical autophagy and degradation.
- What is the role of human autophagy in infection and other disease states? It is critical to functionally validate human genetic studies that implicate the autophagy machinery in infectious, autoimmune and inflammatory disease states, and determine if these are diseases of canonical autophagy.
- Unlike bacterial effectors that enable evasion of autophagic recognition (e.g. *Listeria* ActA and InlK, *Shigella* IcsB and VirA, *Salmonella* SseL), a bacterial effector that targets the autophagy machinery for intracellular survival has recently been discovered (Choy *et al*., 2012). What specific mechanisms have bacterial pathogens evolved to inhibit autophagy?
